# Comparison of soil and corn residue cutting performance of different discs used for vertical tillage

**DOI:** 10.1038/s41598-021-82270-9

**Published:** 2021-01-28

**Authors:** Zhiwei Zeng, Dillon Thoms, Ying Chen, Xu Ma

**Affiliations:** 1 College of Engineering, South China Agricultural University, Guangzhou, Guangdong China; 2 Department of Biosystems Engineering, University of Manitoba, Winnipeg, MB Canada; 3 Guangdong Laboratory for Lingnan Modern Agriculture, Guangzhou, Guangdong China

**Keywords:** Ecosystem ecology, Agroecology

## Abstract

High amount of corn (*Zea mays* L.) residue left in the field interferes with seeding operations, which hinders the viability of conservation agriculture. Vertical tillage is a promising practice in dealing with heavy crop residue, but its effectiveness largely depends on the design and use of tillage machines. In this study, three vertical tillage discs with different shapes, namely notched, plain, and rippled, were tested in a soil bin at two different working depths, shallow (63.5 mm) and deep (127 mm). Corn residues were spread on top of the soil as surface residue. soil cutting forces, soil displacement, and residue mixing with soil, as well as residue cutting were measured. The results showed that the working depth had a stronger effect on the performance of discs as compared to the disc type. No difference in residue cutting was found between the treatments. The deep working depth resulted in 5.1% higher residue mixing, 53.4% greater soil cutting forces, and 34.9% larger soil displacements, as compared to the shallow depth. The rippled disc resulted in the largest soil displacements with the greatest demand in soil cutting forces. Overall, the rippled disc was the most aggressive among the three discs with regard to the performance indicators measured. The results suggested that varying working depth would be an effective approach in changing the soil dynamics and residue cutting performance of the discs for vertical tillage.

## Introduction

Conservation agriculture maintains at least 30% residue cover on the soil surface to reduce soil erosion, conserve soil organic matter, and enhance biodiversity and productivity^1^. Conservation tillage, including no-till, has been widely adopted in recent decades for many crops, but challenges in residue management remain, especially for corn (*Zea mays* L.). Corn production generates a significant amount of residue and corn residue itself is slow in decomposition^2^, which results in accumulated residue remaining in the field. This can cause equipment plugging and poor soil-seed contact for seeding operations, which would negatively affect plant emergence and ultimately crop yield^3,4^. Modern harvesters use adapters to chop and crush the residue. For situations where adapters are not available, it is necessary to employ tillage operation to break up the residue and mix it with soil for faster decomposition. Furthermore, field studies have shown that incorporating corn residue into the soil increased the thousand-kernel weight, grain yields, and nitrogen efficiency of the following crop as compared to tillage treatments without residue biomass return^5^.

Vertical tillage (VT), a newly coined form of tillage practice, can deal with high amount of crop residue in a sustainable manner. The general consensus is that VT refers to the soil loosening in relatively vertical lines, although a precise definition has yet to be established. Discs used in VT are designed to cut crop residue into small pieces and to incorporate them partially into the soil with minimal soil disturbance^6^. Previous studies have shown promising results of the VT in terms of soil and water conservation and crop yield^7,8^. However, little research has been done on the mechanisms of soil dynamics and residue management of VT discs.

The majority of recent studies regarding the performance of disc tillage focused on soil cutting forces^9^, soil disturbance^10^, residue incorporation^11,12^, residue spatial distribution^13^, and fuel consumption efficiency^14^, but not on residue cutting effectiveness. Few earlier studies of residue cutting focused on disc openers for no-till planting and other seeding machines^15–18^. Disc openers for seeding were mainly for slicing through the residue to reduce the possibilities of residue plugging and dragging; whereas discs for tillage involve intensive residue cutting as well as mixing with soil. Assessment of disc performance in the context of VT was much needed and this study was conducted to fill those gaps.

The performance of a disc depends on the design and operational parameters of the disc. One of the disc design parameters is its shape (plain, notched, fluted, and rippled, etc.) and associated cutting edge. Previous studies have shown that the residue cutting effectiveness was significantly affected by disc shape^19^, but there were also contrasting reports where four different disc shapes of smooth, fluted, rippled, and notched had similar residue shearing performance^20^. A toothed disc was developed for working on sugarcane residues, and the soil bin test results showed that the disc had a higher residue cutting efficiency and lower soil cutting force as compared to smooth and notched discs^21^. Field tests have shown that rippled discs were less aggressive in disturbing soil and incorporating residue than fluted discs^12^. As for the operational parameters, travel speed was of importance to the performance of concave powered discs^17^ and fluted discs as well^11^. However, little information on the effect of the working depth of VT discs was available.

In conclusion, VT is a promising tillage practice to deal with high amount of corn residue, and its effectiveness is largely dependent on the design and operational parameters of the discs used. The objectives of this study were to investigate the effects of working depth of different types of discs on (1) soil cutting forces, (2) soil displacements, (3) residue mixing with soil, and (4) corn residue cutting.

## Results

The results of ANOVA tests were summarised in Table 1. None of the interaction effect was significant. Therefore, the main effects of disc type and working depth were presented in the following sections.

**Table 1:** Summary of ANOVA test results.

Factor	*df*	*p*							
		Soil cutting force			Soil displacement			Residue	
		Draft	Vertical	Lateral	Forward	Lateral	Vertical	Mixing	Cutting
Disc	2	0.72	0.04	0.29	0.22	0.006	0.88	0.03	0.06
Depth	1	< .0001	0.008	0.009	0.008	0.01	0.32	0.01	0.10
Disc*Depth	2	0.52	0.54	0.73	0.69	0.22	0.07	0.98	0.25

### Soil cutting forces

**Figure 1:**
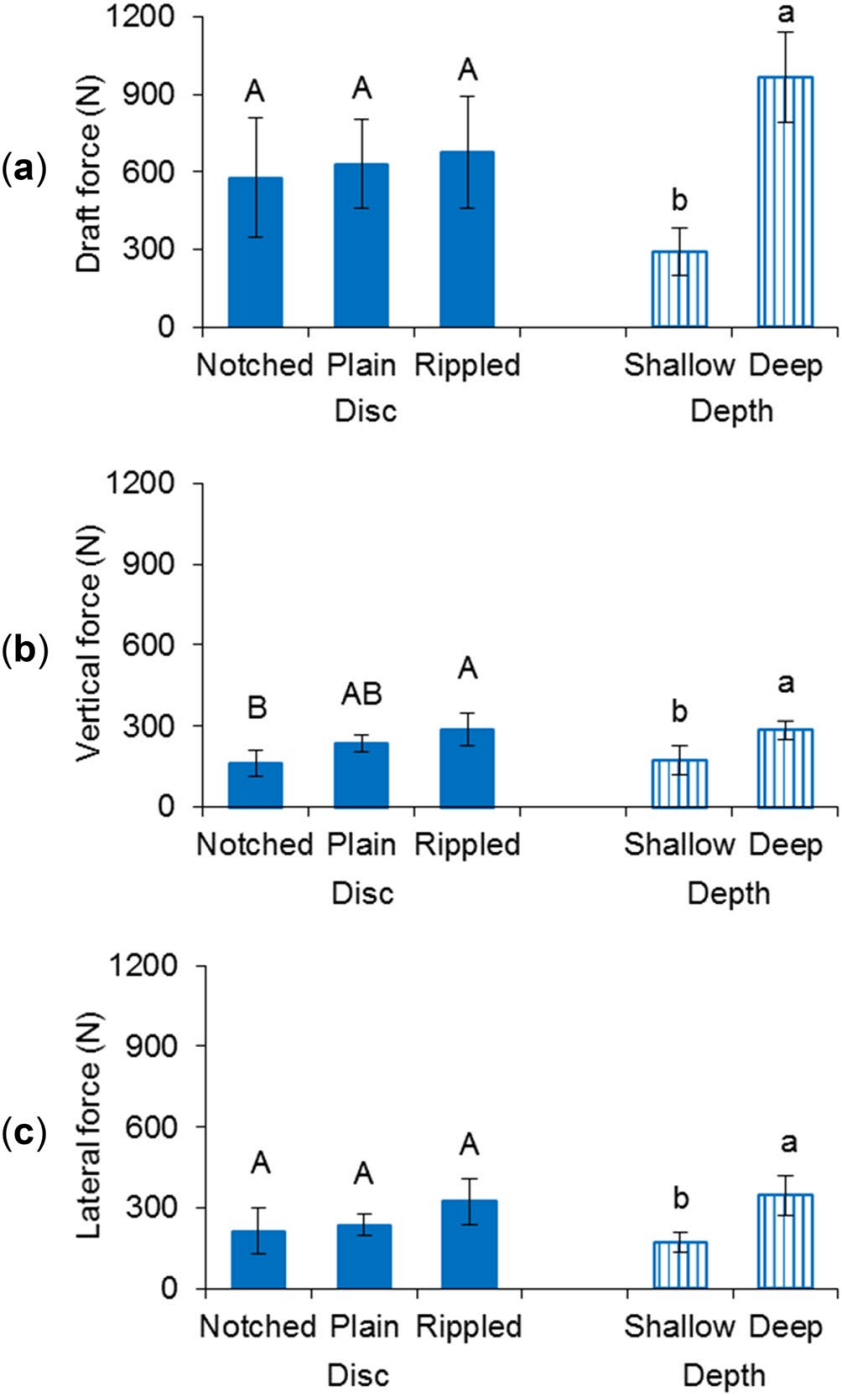
Soil cutting forces of different discs at different working depths: (**a**) draft force, (**b**) vertical force, and (**c**) lateral force; means followed by different lower case letters or upper case letters are significantly different according to Tukey’s test at the significance level of 0.05; error bars are standard deviations.

The rippled disc required an average draft force of 675 N, which was numerically the highest among the discs (Fig. 1a). The notched disc had a minimal draft force demand of 579 N. Increasing the working depth from the shallow (63.5 mm) to deep (127 mm) resulted in the draft force increasing from 291 to 965 N. This more-than-tripled increase was significant and can be explained by the soil dynamics theory that draft force varies with the contact area between soil and tool^22^. The rippled edge slightly increased the surface area as compared to the smooth edge, while the notched edge slightly decreased the contact area due to the notches. As for the depth effect, a deeper operation significantly increased the portion of the disc in contact with soil regardless of the disc type.

All the vertical forces measured were positive, which indicated that they were acting on the disc in the downward direction (Fig. 1b) that favored the disc penetration into the soil. The rippled disc had the maximal vertical force of 289 N, which will help to maintain its working depth as compared to the other two discs. As was expected, the notched disc experienced the minimal vertical force of 164 N, which was lower than that of the rippled disc. The plain disc had a medium vertical force, which was not different from the other two discs. The lower vertical force of the notched disc may not necessarily affect its superior ability of soil penetration. The deep working depth created a 65.9% higher vertical force as compared to the shallow depth. The vertical forces of similar magnitudes were also observed in previous studies, such as approximately 200 N in Nalavade et al.^23^.

There were no significant differences among the discs in terms of the lateral force (Fig. 1c). The notched disc had the minimal lateral force of 215 N. The lateral force increased roughly twofold from 171 to 347 N as the disc was operated from the shallow to deep depths, which was significant. The insignificant difference in the lateral force among the discs was partially attributed to their identical disc angles and similar concavity. Lower lateral forces are usually desired in terms of the frame stability of the implement. The increase of the lateral force as the depth increased indicated a great deal of attention must be paid on the frame strength when designing the disc for deep tillage application.

The soil cutting forces were resultant forces of passive cutting reaction on the concave face and the scrubbing reaction on the convex face for a concave disc^24^. Both the cutting force and scrubbing force acted at some angle between the horizontal and vertical directions. The projected soil cutting force was against the travel direction in the horizontal direction and downward in the vertical direction; on the other hand, the projected scrubbing force is along the travel direction and upward. The resultant draft force was against the travel direction and the resultant vertical force was downward, which was the same as that of the cutting force. This agreed with the literature that the scrubbing force on the trailing convex side of the disc tends to be minor compared to the cutting force on the leading concave side of the disc^22^. However, the soil cutting forces were smaller than those reported in Godwin et al.^25^. The combination of shallow concavity and small disc angle used in this study possibly helped in reducing the soil cutting forces in all three directions. The results agreed with that in Choi and Erback^20^, where the notched disc had the least forces and the forces were more dependent on the working depth than the disc shape.

### Soil displacements

**Figure 2:**
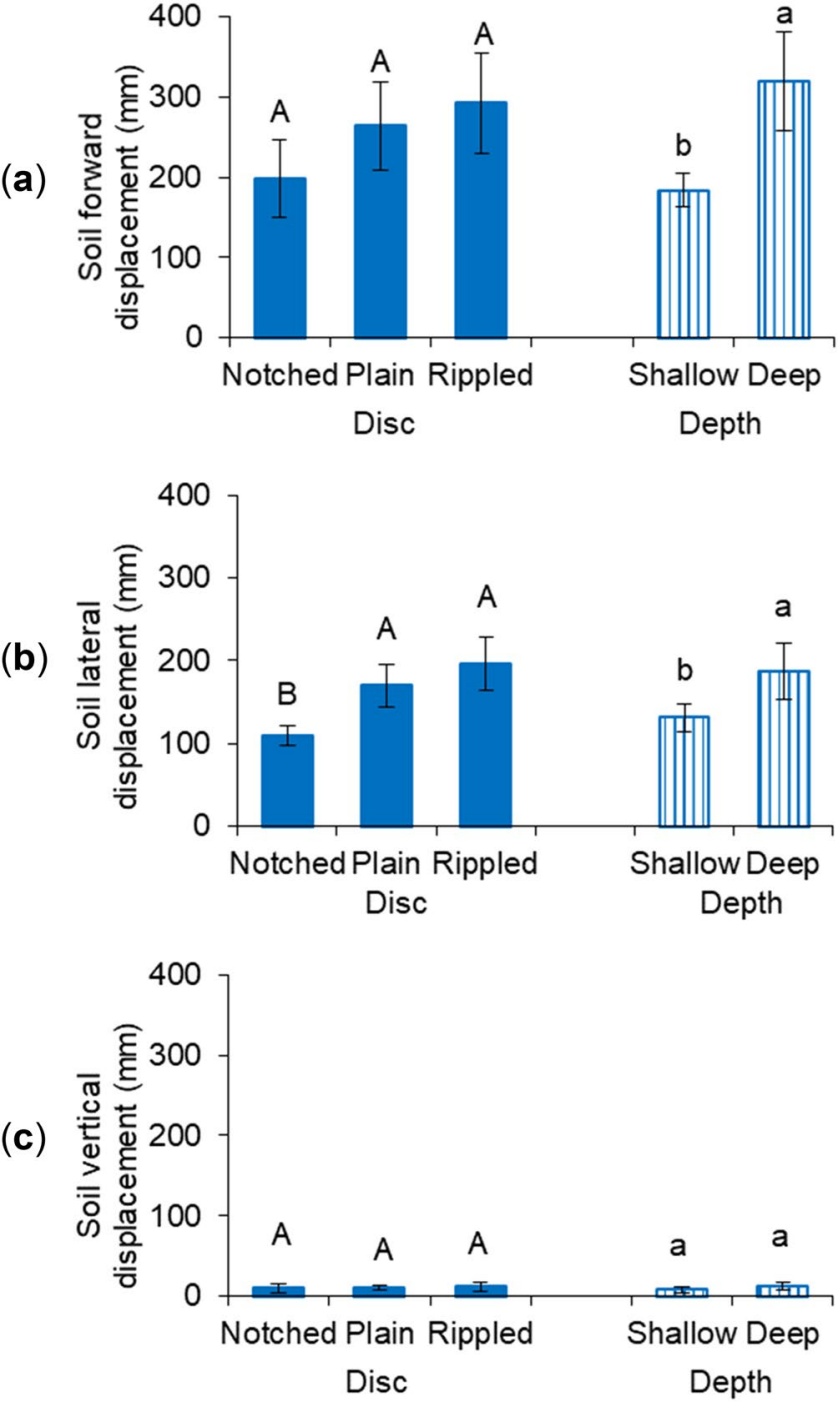
Soil displacements of different discs at different working depths in three directions: (**a**) forward, (**b**) lateral, and (**c**) upward; means followed by different lower case letters or upper case letters are significantly different according to Tukey’s test at the significance level of 0.05; error bars are standard deviations.

The soil forward displacement was maximized with the rippled disc and was minimized with the notched disc (Fig. 2a). The plain disc resulted in a medium soil forward displacement of 264 mm. During the operation of the notched disc, some soil particles might not be pushed forward, but being passed over by the notches. This could explain the small soil displacements observed for the notched disc. However, statistical analysis did not show any significant differences among the three discs with regard to soil forward displacement. The soil tracers were dislodged 184 mm on average when the discs were used at the shallow depth, which was increased by 73.4% at the deep depth.

The rippled disc moved the soil tracers the furthest in the lateral direction at 197 mm (Fig. 2b). The notched disc created the minimal soil lateral displacement of 109 mm, which was less than that of the other two discs. The soil lateral displacement was increased by 42.0% as the working depth changed from the shallow to deep depth. The soil lateral displacement was the average displacement of all the tracers in the lateral direction and a positive value denoted the direction pointing toward the concave face of the disc. It was worth noting that soil tracers on the convex side tended to be pushed away in the opposite direction as compared to other tracers as observed in the experiment. This was related to the scrubbing action as described above.

No significant difference was found in the soil vertical displacement among the treatments (Fig. 2c). All the soil vertical displacements were less than 20 mm with an average of 10.6 mm. Similar to the lateral displacement, not all tracers were dislodged in the same direction. However, the majority of them were in an upward direction including the average value. The small upward displacements indicated moderate soil swelling and elevating movements and minimal soil overturning effect of the discs. This was supported by the soil failure pattern study in Nalavade et al.^23^, which observed that the dominating compressive shear failure pattern of the free-rolling disc discouraged soil inversion actions.

### Residue mixing

**Figure 3:**
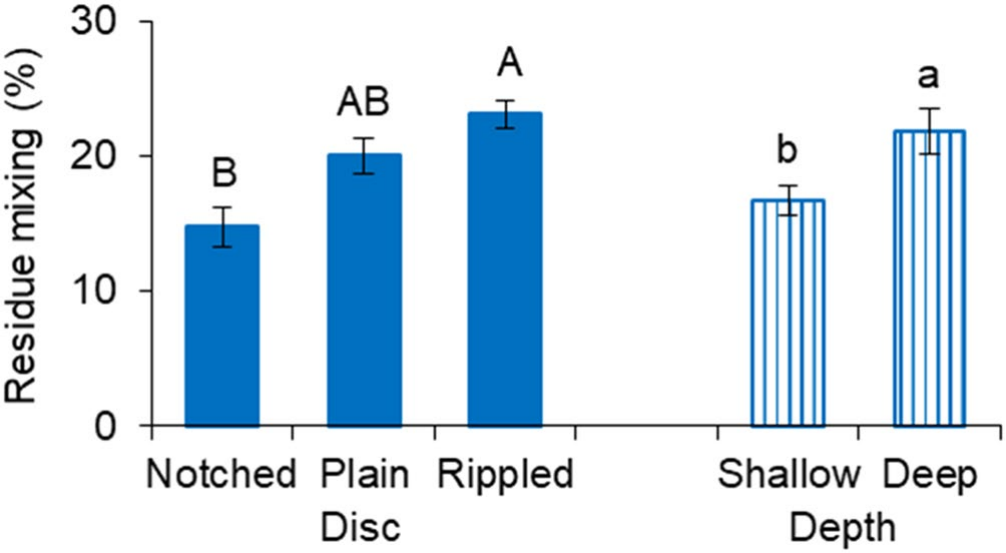
Residue mixing of different discs at different working depths; means followed by different lower case letters or upper case letters are significantly different according to Tukey’s test at the significance level of 0.05; error bars are standard deviations.

The rippled disc had the highest residue mixing rate of 23.1%, which was higher than that of the notched disc, being the lowest at 14.7% (Fig. 3). The residue mixing of the plain disc was medium among the three discs. As for the working depth, the shallow depth created a residue mixing of 16.7%, which was lower than that of the deep depth.

The residue mixing could be used to estimate the amount of residue being incorporated into the soil, given the surface residue before tillage was 7500 kg/ha. Therefore, the rippled disc was the most effective in terms of the residue incorporation at a rate of 2746 kg/ha. The residue incorporation increased by 606 kg/ha as the working depth increased from shallow to deep. Also, deducting the residue mixing from the original residue cover of 63.1% would be the residue cover remaining. None of the treatments resulted in a residue cover less than 30%, which suggested that all treatments would satisfy the requirement of conservation tillage.

### Residue cutting

**Figure 4:**
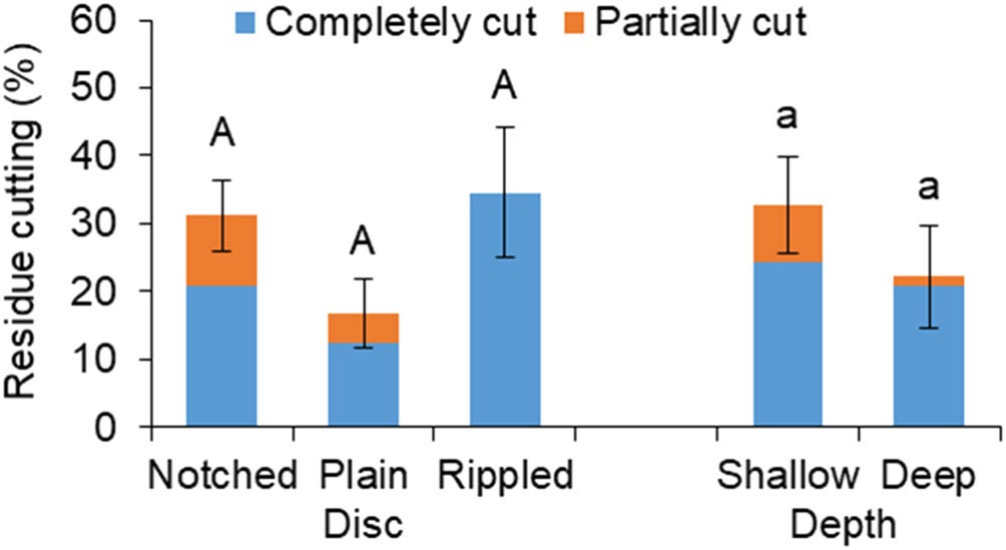
Residue cutting including completely cut and partially cut of different discs at different working depths; means followed by different lower case letters or upper case letters are significantly different according to Tukey’s test at the significance level of 0.05; error bars are standard deviations.

The residue cutting effectiveness of the discs varied from the highest to the lowest as the rippled, notched, and plain with no significant differences were found (Fig. 4). The total residue cutting of the notched disc consisted of one-third of partially cut while no partially cut was observed for the rippled disc. As for the plain disc, roughly a quarter of the total residue cutting was partially cut. The shallow working depth had a numerically higher residue cutting rate than the deep depth: 32.8% versus 22.2%. One in every four residue tracers being cut was partially cut when the discs were operated at the shallow depth. As a comparison, less than one residue was partially cut for every ten residue tracers being cut at the deep depth. The results suggested that the most effective treatment in cutting residues was the rippled disc at the shallow depth. On average, only 27.5% of the residue tracers were being cut, either partially or completely, by the discs. Partial cuts tended to be pushed into the soil and damaged by the discs. The majority of the remaining residue was pushed aside by the discs through disturbed soil.

The effects of disc type and working depth on the residue cutting efficiency of the discs differed from the previous studies of disc openers. For example, the plain disc was found to have a much higher residue cutting efficiency than the notched and serrated discs and the efficiency increased as the working depth increased^17^. The primary cause of the difference was due to the difference in residue cutting mechanism between the angled tillage discs and relatively straight disc openers. The concaved discs disturbed a fair amount of soil ahead of the disc and relied on the edge to “hook” lying residues in order to cut them. Therefore, the rippled and notched discs had numerically higher residue cutting rates than the plain disc thanks to their hooking edges. The shallower the working depth, the less the soil disturbance and the higher the residue cutting efficiency is. On the other hand, a straight disc opener would ride over all possible residues on the path and penetrate the soil without causing significant disturbance to the seedbed. The difference in residue cutting effectiveness can also be accounted for in part by the difference in residue characteristics such as type, percent cover, and moisture content. For instance, wet rice residue with a moisture content of 41.4% at 2000 kg/ha^15^ versus dry corn stalk with a moisture content of 4.5% at 7500 kg/ha in this study. Previous studies have shown that the cutting performance of the disc openers was significantly affected by the mechanical properties of the residue^26^ and residue density^17^.

The numerically higher portion of surface residue being cut at the shallow depth was attributed to a smaller cutting angle. This cutting angle was the angle of absolute velocity vector acting on the residue with the vertical axis in Kushwaha et al.^16^, whose analytical model showed that the angle of absolute velocity vector for a disc is smaller at a shallower depth. The disc tended to cut or bend the residues at a smaller cutting angle, while the disc tended to push the residue ahead at a larger cutting angle. The notched disc had the numerically highest portion of partially cut among the three discs, which agreed with the results in Bianchini and Magalhaes^21^. Kushwaha et al.^17^ also observed that residue pieces were held into the notches and serrations of the discs instead of being cut, being thrown backward as the disc exited from the soil.

## Discussion

In general, the effect of working depth was more pronounced than that of disc type in determining the resultant soil and residue conditions. Out of all eight measurements, the working depth significantly affected most of them except for the soil vertical displacement and residue cutting rate. The working speed was found to be more influential than the geometrical parameters of similar VT discs in an earlier work of Zeng and Chen^11^. Therefore, it was fair to draw that the operational parameters were more dominated than the design parameters of VT discs in terms of soil dynamics and residue management performance. The deep working depth was greater than the shallow one by a factor of two. Halving working depth reduced the soil cutting forces and soil displacements by an average 53.4% and 34.9% respectively, which indicated non-linear impacts of the depth. Soil cutting forces and soil displacements had similar trends in response to the variation in the treatments. This was consistent with the results in Hasimu and Chen^27^. As expected, the soil displacements were reversely correlated to the residue cover remaining. The greater the soil displacements were, the more the residue being incorporated, and therefore, the less the residue cover would remain on the soil surface.

The information gained in this study is important for the design and use of VT disc implements for corn residue management. The results supported the general perception of the purpose of different discs and also quantified the differences in the performance of these new VT discs. The rippled disc was the most aggressive in terms of both disturbing soil and cutting residue, which resulted in the least amount of residue remaining on the surface. The notched disc required the least amount of force to pull and was the second-best in residue cutting with a significant portion of the partially cut. The plain disc was intermediate for the majority of measurements except for the residue cutting rate, where it was the least effective among the discs. The results have many other implications. For example, there is no different between the notched and rippled disc in terms of residue cutting. However, the rippled disc had a greater vertical force, which would indirectly affect the residue cutting ability of a VT implement. The insufficient downward force applied to disc-type implement would result in the surface residue being pushed into the bottom of the furrow instead of being cut^28^. Despite the difference between discs, it would be more effective by varying working depth to alter tillage performance as compared to using different discs. Corn residue in the field can be classified into three different types including standing stubbles, lying stalks, and leaves, among which detached lying stalks presented the most difficult residue handling problem for seeding operations^29^. The use of lying stalks in the experiment was justified on this basis. However, cautions must be exercised in applying the findings to a field condition where standing stubbles present and the moisture content of residue differs. The discs were tested in a single-shank configuration, which would provide superior performance in uneven and rocky fields with the ability to handle high impact loads. However, one should expect to see an extra manufacturing cost associated with individual shanks as compared to gang configurations.

## Conclusion

The performance of three different discs for vertical tillage was assessed at two working depths by measuring soil cutting forces, soil displacements, residue cutting, and residue mixing. Dry corn stalk was used in the experiment. The results showed that the working depth was more pronounced than the disc type in affecting tillage performance. Increasing the working depth resulted in increases in soil cutting forces, soil displacements, and residue mixing. As for the disc type, the rippled disc demanded the highest vertical force of 289 N while the notched disc had the lowest values. Similarly, soil lateral displacement was maximized with the rippled disc and minimized with the notched disc. The difference in residue cutting was not significant to the working depth nor the disc type. The residue mixing rate varied from low to high as notched, plain, and rippled. Among the three discs tested, the rippled disc was the most effective tool in mixing them into the soil. The findings would serve as references when designing and selecting tillage implement of conservation agriculture.

## Methods

### Description of the discs

**Figure 5:**
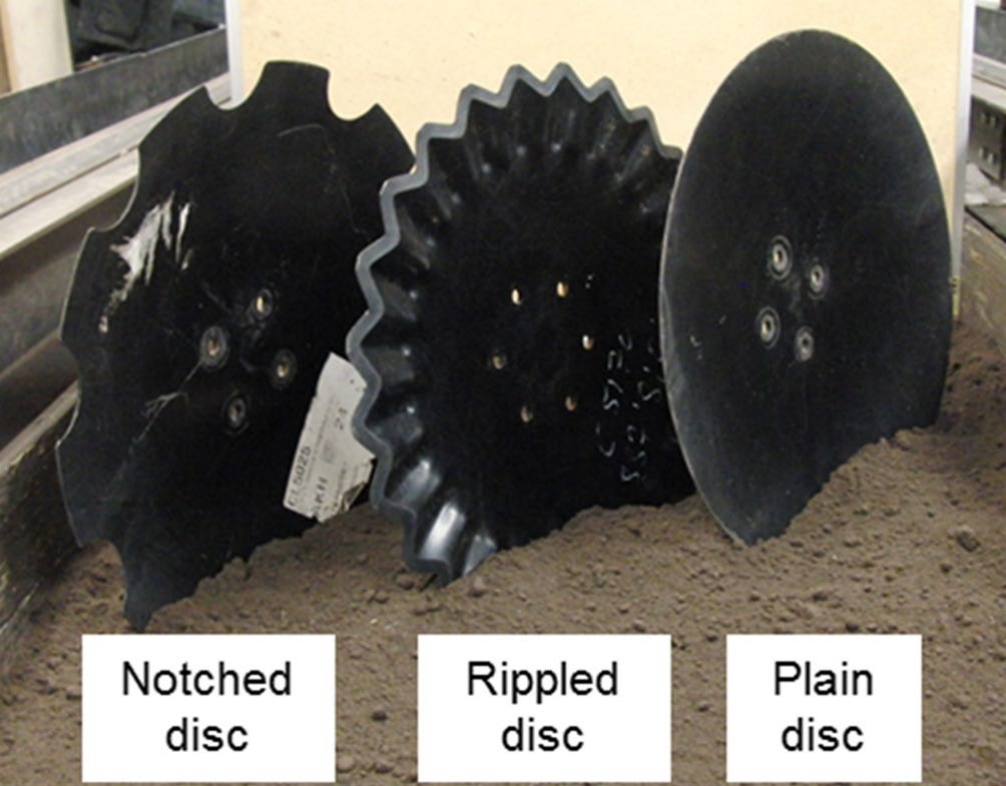
Three discs tested with different shapes: notched, rippled, and plain.

Three discs with different shapes including notched, rippled, and plain were tested in this study (Fig. 5). The plain disc had a smooth and beveled cutting edge. The notched disc had 10 notches evenly distributed along the circumference; each notch had an opening of 79 mm at the periphery and a depth of 29 mm in the radial direction. The rippled disc (SoilRazor VT, Ingersoll Tillage Group Inc.) featured a continuous sawtooth wave design. The edge of the rippled disc was composed of a total of 25 waved teeth; each tooth had a height of 13.5 mm and a peak and valley length of 80.0 and 60.0 mm in the radial direction. All disc blades had a sharpened circumference. The notched disc was known for its soil penetration ability. The rippled disc was designed to slice and size the crop residues while self-sharpening. The plain disc was the most versatile one and commonly used in general conditions. The discs had the same thickness of 6.5 mm and the same diameter of 508 mm. All discs were concave discs with similar shallow concavities (34 mm for the notched and the plain; 30.5 mm for the rippled).

### Testing facility and setup

**Figure 6:**
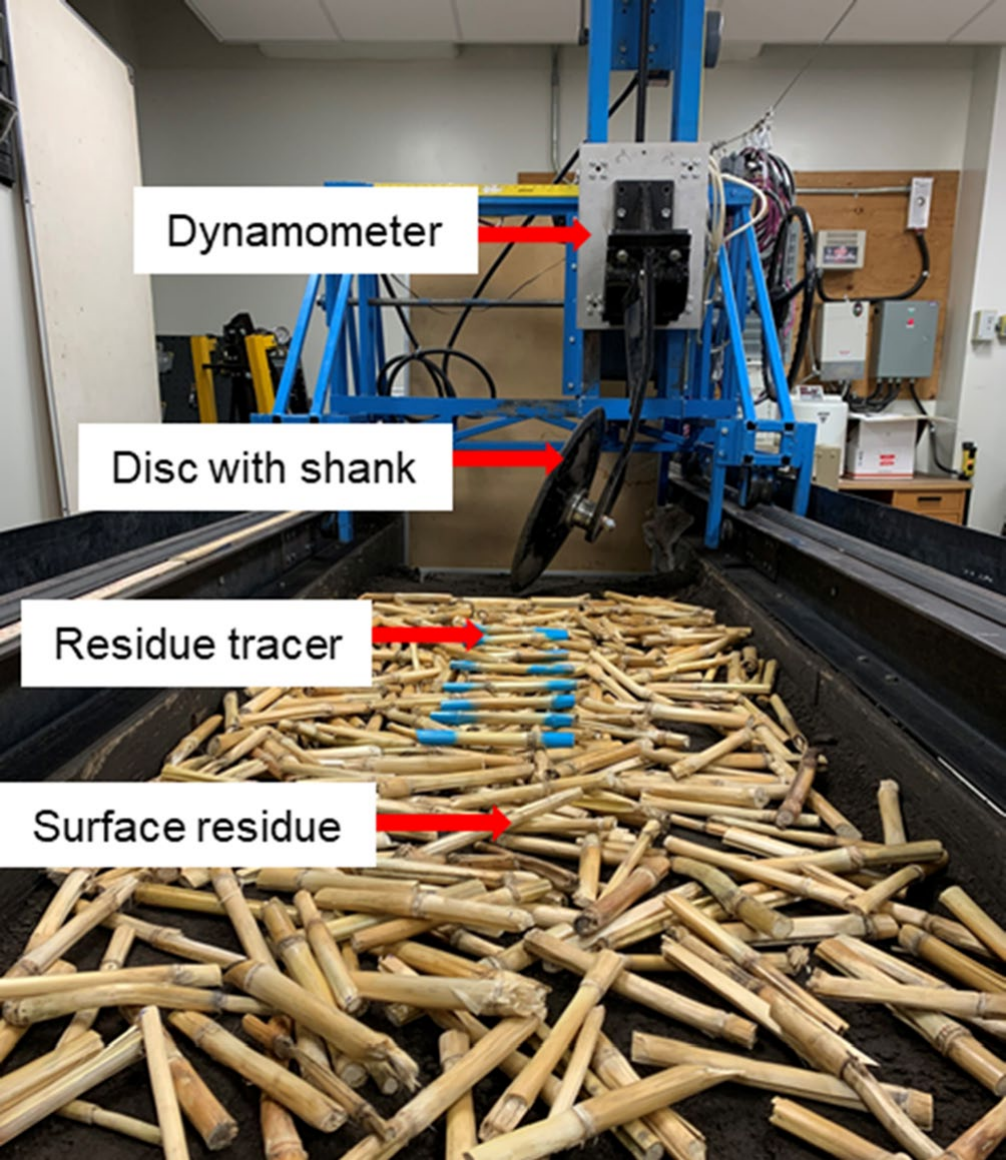
The testing facility showing the soil bin, corn residues prior to the test, a disc and shank mounted on the soil bin carriage through a dynamometer.

The discs were tested in an indoor soil bin at the Soil Dynamics and Machinery Lab, University of Manitoba, Canada. A curved rigid shank was designed and fabricated to mount the discs individually on the soil bin carriage (Fig. 6), which set the disc at a disc angle of 16 degrees relative to the direction of travel and a tilt angle of 20 degrees from vertical. The discs were tilted in a direction such that the centre of the disc sphere was above the centre of the disc. Disc and tilt angles have been known for affecting tillage performance^30–32^. For example, typical values of disc and tilt angles ranging from 10 to 30 were investigated on their effects on soil cutting forces in Sadek et al*.*^33^. The angles were arbitrarily chosen within the typical ranges in the literature. The soil bin was 10 m in length, 0.9 m in width, and 0.6 m in depth. The bin was filled with a sandy loam soil (70% sand, 16% silt, and 14% clay). The soil was prepared using a standard procedure consisted of loosening, leveling, and compacting prior to each test to ensure a consistent soil physical condition^10^. The soil condition was monitored throughout the test by taking a total of 18 soil core samples (50 mm in diameter and 100 mm in depth). The samples were weighed, oven-dried at 105ºC for 24 h, and weighed again to determine soil moisture content and bulk density^34^. Soil penetration resistance at the surface was measured using a Pocket Penetrometer (Geotester, Italy) at 18 random locations throughout the tests. The average soil moisture content with standard deviation was 17.0% ± 1.2% (d.b.) and the average dry bulk density with standard deviation was 1.48 ± 0.11 g/cm^3^, which was typical for the soil tested. The soil surface penetration resistance with standard deviation was 752 ± 98 kPa.

### Experimental design

The experiment was conducted using a completely randomized factorial design. Two factors of the disc type and working depth were examined. Three different discs (notched, rippled, and plain) were combined with two different depths (shallow and deep), with three replicates, giving a total of 18 test runs. The operating depth for shallow and deep tillage was 63.5 and 127 mm respectively. All discs were tested at a constant speed of 10 km/h.

### Measurements

#### Soil cutting forces

A plate dynamometer was mounted between the disc shank and the soil bin carriage to measure the soil cutting forces of the disc (Fig. 6). The dynamometer was designed and calibrated to independently measure the forces in three perpendicular directions, namely, draft, vertical, and lateral forces^35^. Force signals were recorded using a data acquisition system at 65 Hz.

#### Soil displacements

**Figure 7:**
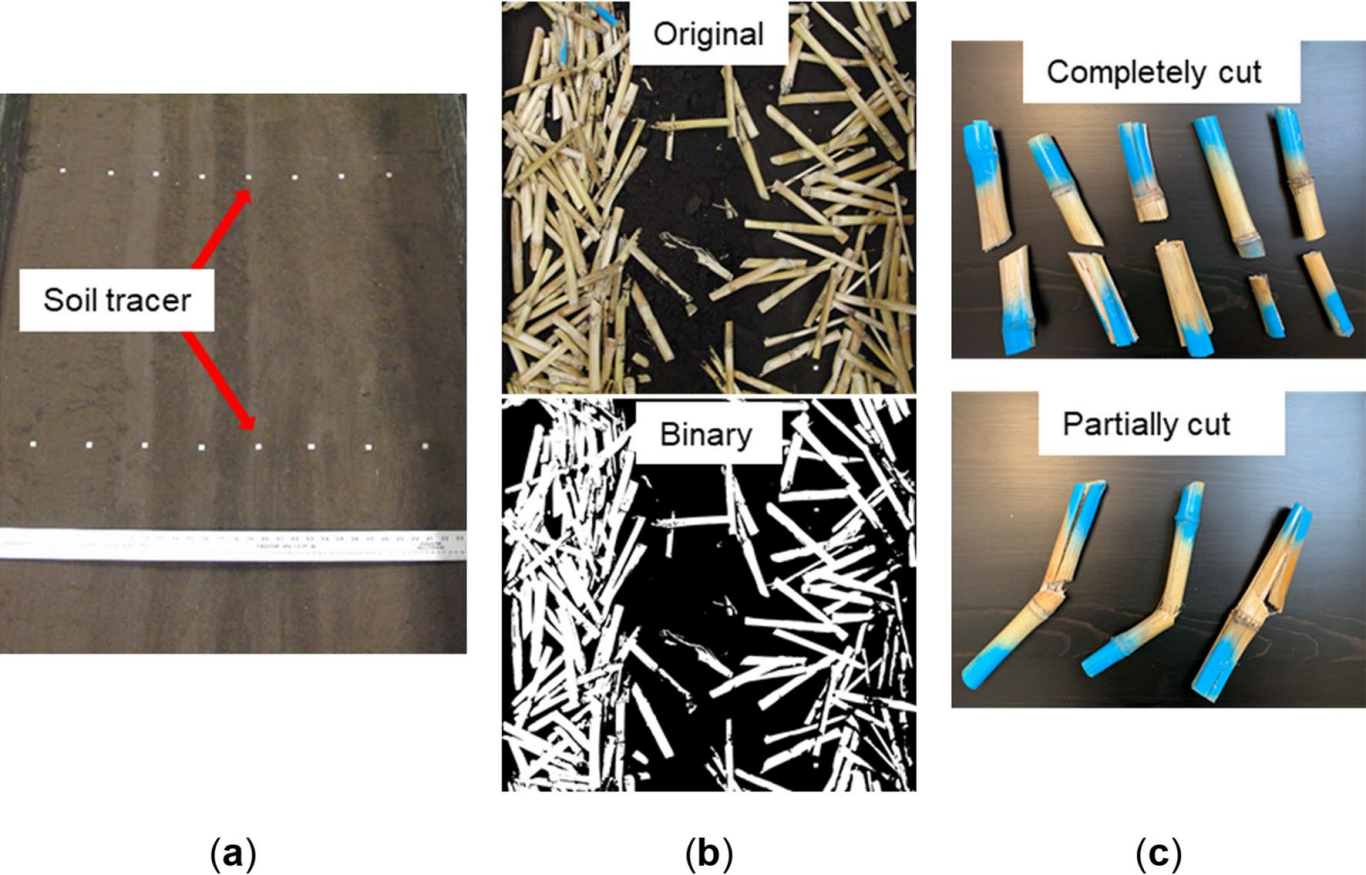
Soil bin test measurements: (**a**) soil tracers used for monitoring soil displacements, (**b**) original image and binary image for measuring residue mixing, (**c**) sample residue tracers demonstrating two different residue cutting scenarios, completely and partially cuts.

Soil displacement was measured using aluminum cubic tracers of 10 mm in side length. A total of 16 numbered tracers were arranged in two rows perpendicular to the travel direction and in the middle section of the soil bin (Fig. 7a). The distance between adjacent tracers in a row was 100 mm. The tracers were inserted into the soil until their top face flushed with the soil surface and their initial coordinates were recorded. After the tool passage, the tracers were carefully located and their final coordinates were recorded again. The differences in the initial and final coordinates in a direction were defined as the soil displacements of the perspective direction.

#### Residue mixing

Corn stalks were collected from a farm after harvesting (moisture content: 4.5% ± 0.4%) and cut into uniform segments of 200 mm. Each segment included a randomly located internode. Only the stem part of the corn plant residue was used in the experiments. A total of 1350 g residue was randomly spread over a 2-m long soil surface in the middle section of the soil bin to create an equivalent residue cover of 7500 kg/ha as measured in Zeng and Chen^12^ (Fig. 6). The surface residue was prepared with an intention to best represent the typical field condition that the discs would encounter in a corn field. Ideally, the residue mixing should be measured by weighing incorporated residues in the soil resulting from tool passage. However, partial burials of corn stalks were observed, which would cause difficulties in deciding which stalk to weigh or not. Therefore, the extent of residue mixing was indirectly measured by quantifying the reduction of residue cover through the image analysis method^8^.

The soil bin surface images were taken both before and after tillage operations and a thresholding method was implemented in Matlab to convert them into binary (black-and-white) images with white pixels being residue and black being soil (Fig. 7b). The threshold value differentiating soil and residue was based on their pixel colors by a trial and error method. The residue cover was then calculated as the percentage of the number of white pixels to the total pixels of an image. The residue cover before the tests was 63.1% on average. The residue mixing was calculated as the residue cover reduction.

#### Residue cutting

A total of 8 residue pieces were painted in blue at two ends, and used as residue tracers to monitor residue cutting effectiveness. The residue tracers were orientated along the soil bin width direction and were placed 100 mm apart in the travel direction (Fig. 6). Two different residue cutting scenarios: completely and partially cut, were recorded. The completely cut referred to a residue tracer being cut into two separated parts while the partially cut referred to the residue remained a single piece while being partially cut as illustrated by sample tracers in Fig. 7c. A set of intact residue tracers was used for each test. The residue cutting effectiveness was calculated as the percentage of the number of residue tracers being cut (both completely and partially) to the total amount of residue tracers in each test.

### Data analysis

Two way analysis of variance (ANOVA) of test results was performed using PROC ANOVA in statistical software (SAS for Windows v 9.4). The factors being tested were disc type, depth, and their interaction. When ANOVA results indicating a significant difference among treatments, the means of each measurement between the treatments were compared with Tukey’s studentized range test at the significance level of 0.05. The main effects of disc type and working depth were presented when the interaction effects were not significant.
